# Metagenomic insights into mechanisms of coral larval settlement induction and inhibition by marine biofilms

**DOI:** 10.1186/s40793-026-00945-0

**Published:** 2026-08-23

**Authors:** Paul A. O’Brien, Sara C. Bell, Andrew P. Negri, Shannon R. Kjeldsen, Julian Zaugg, Nicole S. Webster, Muhammad Abdul Wahab, Inka Vanwonterghem, Laura Rix

**Affiliations:** 1 Australian Centre for Ecogenomics, School of Chemistry and Molecular Biosciences, The University of Queensland, St Lucia, QLD Australia; 2 Australian Institute of Marine Science, Townsville, QLD Australia; 3 Marine Climate Change Unit, Okinawa Institute of Science and Technology, Onna-Son, Okinawa, Japan; 4 Institute for Marine and Antarctic Studies, University of Tasmania, Hobart, TAS 7001 Australia

**Keywords:** Settlement, Coral, Biofilm, Metagenomics, GABA, Secretion systems, Nitric oxide, Restoration

## Abstract

**Background:**

Biofilms are essential to larval settlement in many marine invertebrates, yet the mechanisms driving settlement induction or inhibition in corals remain poorly resolved. This challenge lies in the vast taxonomic and functional diversity of marine biofilms, making it difficult to identify cues associated with settlement. To address this, we analysed the metagenomes of biofilms used to induce settlement (attachment and metamorphosis) of four broadcast-spawning non-acroporid coral species: *Dipsastrea favus*, *Platygyra sinensis*, *Echinophyllia aspera* and *Porites lobata.* Biofilms were developed for one or two months, under light or dark treatments, with light biofilms inducing significantly higher settlement than dark biofilms.

**Results:**

Gene composition varied strongly among treatments, with light biofilms enriched in genes encoding carotenoid biosynthesis and nitrate reduction, while dark biofilms encoded more genes for denitrification and nitric oxide production. Modelling revealed the abundance of genes encoding GABA biosynthesis and the type III secretion system (SS) were positively associated with settlement, while genes encoding the type II secretion system, flagellar and lipopolysaccharides were negatively associated. Genes predicted to promote settlement were concentrated in metagenome assembled genomes (MAGs) assigned to *Flavobacteriaceae*, *Rhodobacteraceae* and *Pirellulaceae*, consistent with previous research identifying these lineages as potential inducers. While we detected homologues of some biosynthesis genes for the settlement-inducing compounds cycloprodigiosin and tetrabromopyrrole in the MAGs, pathways were incomplete suggesting additional compounds promote settlement on these biofilms.

**Conclusions:**

These findings link biofilm metagenomics to coral larval settlement for the first time, suggesting carotenoids may attract larvae to biofilm surfaces, while GABA may promote searching and attachment. Additional compounds, for example cycloprodigiosin, tetrabromopyrrole or effector proteins, may be required to complete metamorphosis, however the specific compounds responsible likely vary across biofilm communities and suggest multiple mechanisms can lead to settlement. Simultaneously, elevated levels of nitric oxide, type II SS exudates or an abundance of flagellar potentially inhibit the settlement process. This study advances our understanding of the complex microbial processes underpinning coral larval settlement.

**Supplementary Information:**

The online version contains supplementary material available at https://doi.org/10.1186/s40793-026-00945-0.

## Background

Marine biofilms are often complex yet structured microbial communities, characterised by high taxonomic diversity and vast functional potential [1]. They colonise a wide variety of benthic substrates, providing a dynamic living surface that interacts with surrounding marine life [2]. One important interaction is their capacity to influence larval settlement, with outcomes ranging from facilitating coral reef recruitment to biofouling on ship hulls [3]. The significance of biofilms in larval settlement (here defined as both attachment and metamorphosis [4]) has been recognised for decades [5], spanning numerous marine invertebrate phyla and leading to the discovery of novel bacterial functions [6]. However, the mechanisms by which biofilms mediate coral larval settlement remain poorly understood.

Inducing coral larval settlement in aquaculture is essential for studying early life history and for scaling up coral restoration efforts. Settlement is often achieved using crustose coralline algae (CCA) harvested from reefs or by conditioning substrates in aquaria or the ocean to allow biofilms to develop [7]. However, both approaches present limitations: CCA harvesting from reefs can be destructive, and effectiveness varies across coral species [8, 9], while substrate conditioning requires months and often yields inconsistent outcomes [7]. Furthermore, many non-acroporid species remain particularly difficult to settle, hindering their inclusion in restoration programs. To overcome these challenges, innovative settlement-enhancing technologies are being explored, including Bacterial Reef Ink (Brink) and SNAP-X, which can be applied directly to substrates to improve larval settlement [10, 11]. Improving our understanding of the microbial mechanisms and taxa driving settlement cues will be critical for further developing such innovations, enabling more reliable and scalable settlement strategies for coral research and restoration.

Understanding how biofilms induce coral larval settlement remains challenging. A wide range of bacterial taxa show inductive potential, yet these taxa follow no phylogenetic pattern, nor do they provide a consistent mechanism of induction [12]. For instance, bacterial species from different taxonomic classes may strongly induce larval settlement, while others within the same genus elicit no response [13, 14]. Even within a single genus, distinct mechanisms have been described. For example, in *Pseudoalteromonas,* settlement induction can occur through the biosynthesis of tetrabromopyrrole (TBP) or through light degradation of cycloprodigiosin [15, 16]*.* These metabolites can elicit varying responses across coral species; TBP for example, strongly induces settlement in some coral species but triggers metamorphosis without attachment in others [17, 18]. Currently, TBP and cycloprodigiosin remain the only two bacterial metabolites characterised as settlement inducers, but given the complexity of marine biofilms, encompassing a diverse range of taxa and metabolic compounds, additional mechanisms are highly likely.

Research into bacterial interactions with coral larvae has largely taken two approaches: community-level characterisation of biofilms using 16S rRNA gene amplicon sequencing, and functional investigation focussing on promising bacterial isolates. Amplicon sequencing has provided valuable insights into how coral larvae respond to biofilm microbial ecology. For example, network analysis has shown that biofilm communities transition from low to high settlement induction states with the presence of certain taxa [19], while other studies have demonstrated that community shifts linked to poor water quality can lead to decreased settlement [20, 21]. In contrast, studies with microbial isolates have elucidated mechanistic insights, such as the discovery that light degradation of cycloprodigiosin releases hydrogen peroxide, which in turn stimulates settlement [16]. However, both approaches have limitations that prevent a comprehensive understanding of settlement in situ. While amplicon sequencing provides valuable taxonomic context to microbial communities, it does not address the functional aspect of the community. On the other hand, studying isolates allows for functional investigation, but their behaviour may differ when in an in situ heterogenous biofilm community. Cultivation of isolates is also technically challenging, and most isolates tested to date originate from CCA [13, 22]. To bridge these challenges, biofilm community composition can be linked to functional potential through metagenomics.

In a previous study, we showed that biofilms developed under light (referred to as light biofilms) for two months induced higher rates of settlement than those developed for one month or under darkness (dark biofilms) [23]. Community composition differed markedly among treatments, and settlement success correlated with certain taxa including *Flavobacteriaceae* (Bacteroidetes), *Rhodobacteraceae* (Proteobacteria) and *Pirellulaceae* (Planctomycetes)*.* These results established an important link between biofilm community structure and larval settlement but did not resolve how the biofilm induced settlement. We hypothesised that variation in metabolic function and compound production among biofilms likely influenced settlement of coral larvae. Hence, in this study we sequenced and analysed the metagenomes of biofilms used previously [23] to predict mechanisms of settlement induction and inhibition of non-acroporid coral larvae. Although biofilms also include microbial eukaryotes, our analysis focussed on prokaryotes, with the aim of identifying bacterial mechanisms of induction that could be harnessed for biotechnological applications in reef restoration.

## Methods

### Coral larval settlement in response to biofilms developed under different treatments

For full details of the larval settlement experiment and culturing methods, we refer readers to our previous study [23]. Briefly, experiments were conducted during the 2021 Great Barrier Reef (GBR) mass spawning events using larvae from the non-acroporid coral species *Platygyra sinensis* (Merulinidae)*, Dipsastrea favus* (Merulinidae), *Echinophyllia aspera* (Lobophyllidae) and *Porites lobata* (Poritidae). Biofilms were formed on concrete sheets constructed to enable the generation of smaller tabs (herein referred to as settlement tabs) measuring 14 × 14 mm. Biofilm development occurred separately under both light (photoperiod set to local sunrise and sunset times) and dark (24 h darkness) treatments for two time periods: 1 month (1M) and 2 months (2M). Each light and dark treatment was replicated across three independent tank systems, yielding a total of twelve treatment by tank combinations for biofilm development. Negative controls consisted of unconditioned concrete settlement tabs soaked in 0.1 µm filtered seawater (FSW) for approximately one week, with FSW refreshed three times and sterilised by autoclave.

Settlement assays were conducted using sterile 6-well culture plates (Corning Costar TC-Treated, Merck) in a temperature-controlled room (27–28°C) with a light photoperiod set to match local sunrise and sunset times in Townsville, QLD, Australia. Each well was filled with 10 mL of 0.1 µm filtered seawater (FSW), larvae added (n = 6), followed by a settlement tab. Assays were assessed after ~ 48 h, and larvae were scored as settled if they were firmly attached to either the substrate or well and showing signs of metamorphosis [4]. Following settlement assays, each settlement tab was wrapped in aluminium foil, labelled and placed into a whirl–pak bag (grouped by treatment) and snap frozen in liquid nitrogen.

### DNA extraction and sequencing

Settlement tabs were pooled for each metagenome extraction to ensure sufficient biomass for appropriate DNA yield and metagenome sequencing. Settlement tabs were grouped into categories of no settlement, low settlement (≤ 50% settled) and high settlement (> 50% settled), within each tank and treatment combination for each coral species tested. Samples were pooled into a maximum of four tabs (mean = 3) per metagenome extraction per category. Where possible, replicates with the same proportion of larvae settled were pooled together (Table S1). DNA from biofilms on settlement tabs was extracted using a phenol:chloroform:isoamyl alcohol extraction protocol described in Supplementary File 1.

Metagenomic sequencing (2 × 150 bp) was conducted through the Australian Centre for Ecogenomics (ACE) on an Illumina NovaSeq6000 platform using the NovaSeq6000 SP kit v1.5. Libraries were prepared according to the manufacturers protocol using the Nextera DNA library preparation kit (Illumina # 20060059) with a reduction in total reaction volume for 96-well plate format processing.

### Data pre-processing, assembly and binning

Raw reads were first quality trimmed using fastp (v0.23.2) [24] to remove polyG tails (Novaseq artifacts), adapter sequences and low-quality reads (Q < 15; Table S2). Trimmed reads were used to generate taxonomic profiles using SingleM (v0.13.2) [25] with the GTDB 07-RS207 metapackage and assembled using metaSPAdes (v3.15.3) [26]. To reduce computational time and assembly size, one biofilm replicate per treatment × tank × settlement category (as above) for each coral species was assembled, yielding a total of 73 metagenome assemblies (Table S3). Trimmed reads from each sample were then mapped back to corresponding assemblies using CoverM (v0.6.1) [27] with minimap2 [28], applying a minimum read alignment of 75% and minimum percent identity of 95%. In addition, the proportion of single-copy marker genes in assemblies relative to reads were calculated using the SingleM ‘appraise’ function, to estimate the proportion of successfully assembled reads.

Each assembly was binned using the Aviary ‘recover’ pipeline (v0.5.0; https://github.com/rhysnewell/aviary, unpublished), with reads from samples of the same conditioning treatment and tank used for differential coverage estimation. Briefly, this pipeline involves metagenomic binning using seven binning programs: CONCOCT [29], VAMB [30], MetaBAT [31], MetaBAT2 [32], SemiBin2 [33], MaxBin2 [34] and Rosella (https://github.com/rhysnewell/rosella, unpublished). Recovered bins underwent five iterations of refinement using Rosella’s ‘refine’ function and a non-redundant set of bins across all programs was picked using DASTool [35], followed by another five iterations of refinement using Rosella. Bin quality was assessed using CheckM (v1.1.3) [36] and bins from all assemblies were dereplicated at 95% average nucleotide identity (ANI) using CoverM (v0.6.1), retaining those with a minimum completeness of > 50% and maximum contamination of < 10% to yield a final set of metagenome assembled genomes (MAGs). To assess recovery of MAGs, trimmed reads from each sample were mapped to the final set of MAGs using CoverM (as above), and the proportion of single copy marker genes present in MAGs relative to trimmed reads was evaluated using SingleM (as above), to estimate the proportion of reads successfully binned. MAGs were taxonomically classified and a phylogenomic tree was inferred using the ‘classify’ workflow in the Genome Taxonomy Database Toolkit (GTDB-Tk; v2.1.0) [37] with the GTDB release r207 [38]. Functional annotation was performed using DRAM (v1.3.3) [39] against the Kyoto Encyclopedia of Genes and Genomes (KEGG) database [40].

### Gene-centric profiling

A gene-centric profile of metagenomic samples was obtained by predicting protein coding sequences from each assembly using Pyrodigal (v2.0.2) [41, 42] and extracting all complete genes (i.e., those with start and stop codons) using mfqe (v0.5.0; https://github.com/wwood/mfqe, unpublished). Extracted gene sequences from all assemblies were clustered at 100% protein identity using CD-HIT (v4.8.1) [43] to generate a non-redundant set of genes across all samples. Gene sequences were annotated using DRAM (v1.4.6) ‘annotate_genes’ against the KEGG database. Trimmed reads from each sample were aligned to the final genes catalogue using DIAMOND blastx (v2.0.14) [44] and read counts per gene were normalised to reads per kilobase million (RPKM). To standardise read counts across samples, trimmed reads were aligned to a set of 59 single-copy ribosomal marker genes within the SingleM (v1.0) database and counts were converted to RPKM. Finally, the RPKM values for genes extracted from the metagenomes were divided by the mean RPKM across the 59 single-copy ribosomal marker genes to give a normalised (n)RPKM value for each gene in each sample. Unless otherwise stated, all following analyses were conducted in R (v4.2) [45], with extensive use of the packages tidyverse (v2.0) [46], vegan (v2.6–8) [47], MaAsLin2 (v1.18) [48], mixOmics (v6.28) [49], Complex heatmap (v2.20) [50] and KEGGREST (v1.44.1) [51].

### Statistical analysis

To assess if biofilm treatment influenced metabolic potential, non-metric multidimensional scaling (NMDS) based on a Bray–Curtis dissimilarity matrix was calculated from the nRPKM gene abundances following a log (*x* + 1) transformation. Differences in gene composition across treatments, conditioning tanks and settlement categories (none = 0%, low ≤ 50%, high > 50%) were tested using permutational multivariate analysis of variance (PERMANOVA) on the Bray–Curtis dissimilarity matrix, while differences in group dispersion were tested using permutational analysis of multivariate dispersions (PERMDISP). Gene diversity in each sample was calculated using Shannon’s diversity index, followed by an analysis of variance (ANOVA) and Tukey’s test with a Bonferroni correction to test for significant differences among biofilm treatments.

To identify genes that encode pathways or proteins that might affect settlement, we extracted KEGG-annotated genes and their abundance in nRPKM for each biofilm sample. Where multiple genes were assigned to the same KEGG orthologue (KO), nRPKM values were summed to give a single value per KO. We then examined which KOs differed in abundance among biofilm treatments to understand how differences in microbial metabolism may affect broad settlement patterns. Multivariate linear models were performed using MaAsLin2 (v1.18), with biofilm treatment as a fixed effect, conditioning tank included as a random effect, and gene abundance data (nRPKM) transformed using the default log transformation (log_2_) to improve linearity. The 2M light treatment was used as the reference group, hence, positive associations represented genes that were more abundant in the 2M light group compared to other treatments, while negative associations were less abundant in the 2M light group.

Associations between KO abundance (nRPKM, log_2_-transformed) and the proportion of corals settled were used to understand which bacterial functions might directly affect settlement. Multivariate linear models using MaAsLin2 (v1.18) were run separately for each coral species due to the potential differences in settlement cues, using the proportion of corals settled as a fixed effect with biofilm treatment and conditioning tank as random effects. Dark treatment biofilms were not included to better understand the cues behind low and high settlement biofilms that did not arise from dark conditioning. For all models (treatment and settlement), *p*-values were adjusted for multiple hypothesis testing using the Benjamini–Hochberg method, with an FDR threshold of 0.25 indicating significance [48]. KOs were filtered to retain those with a minimum abundance of 0.001 nRPKM and a minimum prevalence of 10% across all samples. Model assumptions of normality and homoscedasticity were checked through visual inspection of QQ-plots and residuals vs. fitted plots, showing approximately normal distributions and no evidence of systematic patterns of variance.

Due to the large number of KOs significantly associated with settlement, we additionally identified KOs that contributed most to the shared variance between settlement and biofilm gene composition using sparse Partial Least Squares (sPLS) in the mixOmics (v6.28) R package. KOs were filtered to retain those with a minimum abundance of 0.001 nRPKM and a minimum prevalence of 10% across all samples, as well as to remove KOs with near zero variance. The sPLS parameters were tuned by testing a grid of 5–100 predictors across five components using tenfold cross-validation (10 repeats), with the Mean Absolute Error (MAE) as the selection criterion. These parameters were used to fit the final sPLS model and identify which KOs were the strongest predictors of settlement. KOs that were identified across both methods for each coral were retained to give a final set of KOs associated with settlement. Finally, correlations between the abundance of selected genes of interest and settlement were further analysed using the Pearson correlation coefficient on log₂-transformed data.

### Identification of genes in MAGs

To understand which bacterial taxa might be capable of performing functions identified through gene associations, we searched for genes encoding proteins and pathways with potential for settlement induction or inhibition in the annotated MAGs. Additionally, we searched for genes encoding the biosynthesis of bacterial metabolites known to induce settlement in corals, namely, tetrabromopyrrole (TBP) and cycloprodigiosin. The *bmp* gene cluster was used to identify TBP biosynthesis potential [52], while the prodigiosin cyclisation gene (*PRUB680*) was used for cycloprodigiosin [53]. BLAST (v2.12) [54] was used to create a database of all MAGs followed by a translated nucleotide alignment of the query sequences to the translated database (tblastx). Significant matches for gene homology were determined by a minimum e-value of < 1e−10 and a bit score of > 100, for highly conservative orthologue detection thresholds [55]. Gene presence in MAGs was then visualised using the R packages ‘ggtree’ (v3.12) [56] and ‘ggtree extra’ (v1.14) [57], using the phylogenomic tree inferred with GTDB-Tk (above) and overlaying a heatmap of gene presence in each MAG.

### Cycloprodigiosin gene tree

To assess the homology between genes annotated as *AMGO* in this study and the *PRUB680* gene, we downloaded the Uniprot-RP75 curated protein alignment for fatty acid hydroxylase protein family (PF04116) and the *PRUB680* protein sequence characterised in [53]. We then added the protein sequences for *AGMOs* in this study and the *PRUB680* protein sequence to the Uniprot-RP75 protein alignment using mafft (v7.49) [58]. A phylogenetic tree was inferred from the updated alignment using IQ-Tree (v2.2.2.3) [59], with the best fitting amino acid substitution model (Q.pfam+G4, based on Bayesian Information Criterion) selected using the ‘TEST’ option in ModelFinder [60]. Ultrafast bootstrapping was used with 1000 replicates, and the resulting tree was visualised using ‘ggtree’ (v3.12).

## Results and discussion

**Fig. 1:**
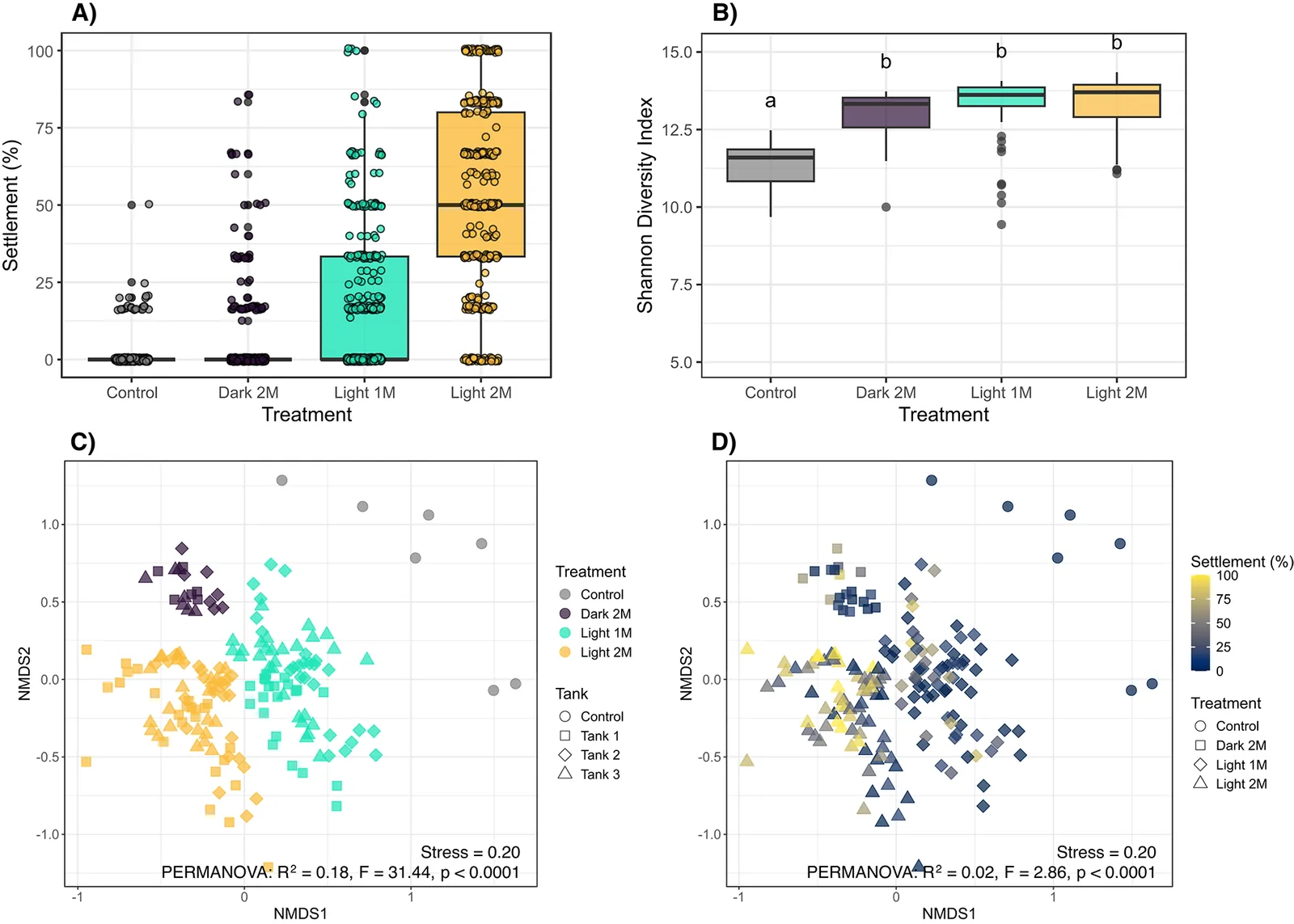
Proportion of corals settled in response to light and dark conditioning treatments (**A**). Boxplots depict settlement of all corals combined (*P. sinensis*, *D. favus*, *E. aspera*, *P. lobata*). **B** Shannon Diversity Index for biofilms in each conditioning treatment based on gene presence and abundance (nRPKM). Letters denote which treatments had a significant difference (*p* < 0.05) in gene diversity based on an ANOVA with a post-hoc Tukey’s test. **C-D** Bray–Curtis dissimilarity among biofilm samples based on gene abundance (nRPKM) visualised using NMDS. Control samples represent unconditioned settlement tabs. 1M and 2M indicate one month and two months conditioning duration. PERMANOVA results for **C** represent treatment as the factor while those for **D** represent settlement

In our previous work, biofilms developed for two months under light induced substantially greater settlement than biofilms developed for one month under light, or biofilms developed in darkness (Fig. 1A) [23]. Here, we build upon these findings using metagenomic analyses of the same biofilms, revealing pronounced differences in the metabolic potential of light compared to dark biofilms that likely underpin these settlement patterns. Within light biofilms, we identified key genes that are associated with larval settlement, suggesting potential mechanisms of both induction and inhibition. This study provides the first metagenomic evidence linking biofilm function to coral larval settlement, greatly improving our knowledge of settlement processes in non-acroporid species.

### Overview of metagenomes and metabolic differences between light and dark biofilms

A total of 173 biofilm metagenomes were sequenced with a depth ranging from 1 to 74 Gbp (mean = 10.5 ± 12.1 SD; Table S2) per sample (excluding blanks and negative control samples). Protein coding sequences predicted from assembled metagenomes resulted in 15.9 million genes, with 8.7 million genes remaining after clustering at 100% sequence identity. Binning of scaffolds yielded 8,212 MAGs, which was dereplicated to 690 MAGs at 95% ANI, with > 50% completion (mean = 82 ± 15.9% SD) and < 10% contamination (2.1 ± 2.0%) (Table S4 & S5). Read mapping to scaffolds estimated that 30–70% (50 ± 10%) of reads were assembled (Table S3), while mapping to the 690 MAGs estimated that 16–47% (33 ± 7.2%) of reads were represented in MAGs (Table S2). Similarly, the proportion of single-copy marker (SCM) genes found in reads that were assembled and binned was estimated at 37–72% (61 ± 6.0%) and 19–44% (33 ± 5.3%) respectively (Table S3 & S2). These results are comparable to other high microbial diversity environments analysed using short-read metagenomic sequencing, such as soil or sediments [62]. However, given the relatively low proportion of reads mapping to the MAGs, we first identified which genes are characteristic of biofilm treatment and coral settlement, and subsequently identified which MAGs encoded these genes to infer the taxa likely influencing coral settlement. Nevertheless, additional taxa with key functional roles in larval settlement may not be captured here.

Taxonomic composition based on SCM genes estimated 114 phyla were present across all biofilm samples, with 20 phyla represented in MAGs. Relative abundance data revealed that Proteobacteria were the most abundant across all samples (mean = 73 ± 6.4% SD), followed by Planctomycetota (7.3 ± 3.0%) and Bacteroidota (4.9% ± 2.9%; Figure S1; Supplementary File 2). At the family level, 1,418 were identified across all biofilms based on SCM genes, while 138 were represented in the MAGs. *Rhodobacteraceae* were the most abundant across all biofilm samples (23 ± 7.9%), followed by *Rhizobiaceae* (4.6 ± 3.4%) and *Hyphomonadaceae* (4.4 ± 2.4%; Figure S2; Supplementary File 2). For a detailed breakdown of taxonomic correlations with settlement we refer the reader to our previous study [23].

While gene diversity did not differ between light and dark biofilms, nor between one- and two-month biofilm development (ANOVA + Tukey’s test; *p* > 0.28; Fig. 1B), gene composition of biofilm samples varied significantly across biofilm treatments based on the nRPKM abundance values (PERMANOVA; *R*^2^ = 0.18; *F* = 31.4; *p* < 0.001; Fig. 1C). This indicates that differences in the types of metabolic functions, rather than the overall diversity of metabolic potential, play a key role in influencing larval settlement. Additionally, smaller differences in biofilm gene composition were detected among conditioning tanks (*R*^2^ = 0.08; *F* = 13.5*; p* < 0.001; Fig. 1C), suggesting tank effects on biofilm development could impact larval settlement, while a weak relationship was found between biofilm gene composition and settlement category (*R*^2^ = 0.02; *F* = 2.86; *p* < 0.001; Fig. 1D). A PERMDISP test revealed significant differences in dispersion among treatment groups (*F* = 30.45; *p* < 0.001), indicating that within-group variability had some influence on PERMANOVA results. However, dispersion did not differ significantly among tanks (*F* = 0.42; *p* = 0.66) or settlement categories (*F* = 0.62; *p* = 0.55), supporting differences in gene composition among these groups. These results are in line with differences in taxonomic composition based on the 16S rRNA gene [23], suggesting that a change in biofilm community composition was paired with a change in biofilm metabolic potential.

**Fig. 2:**
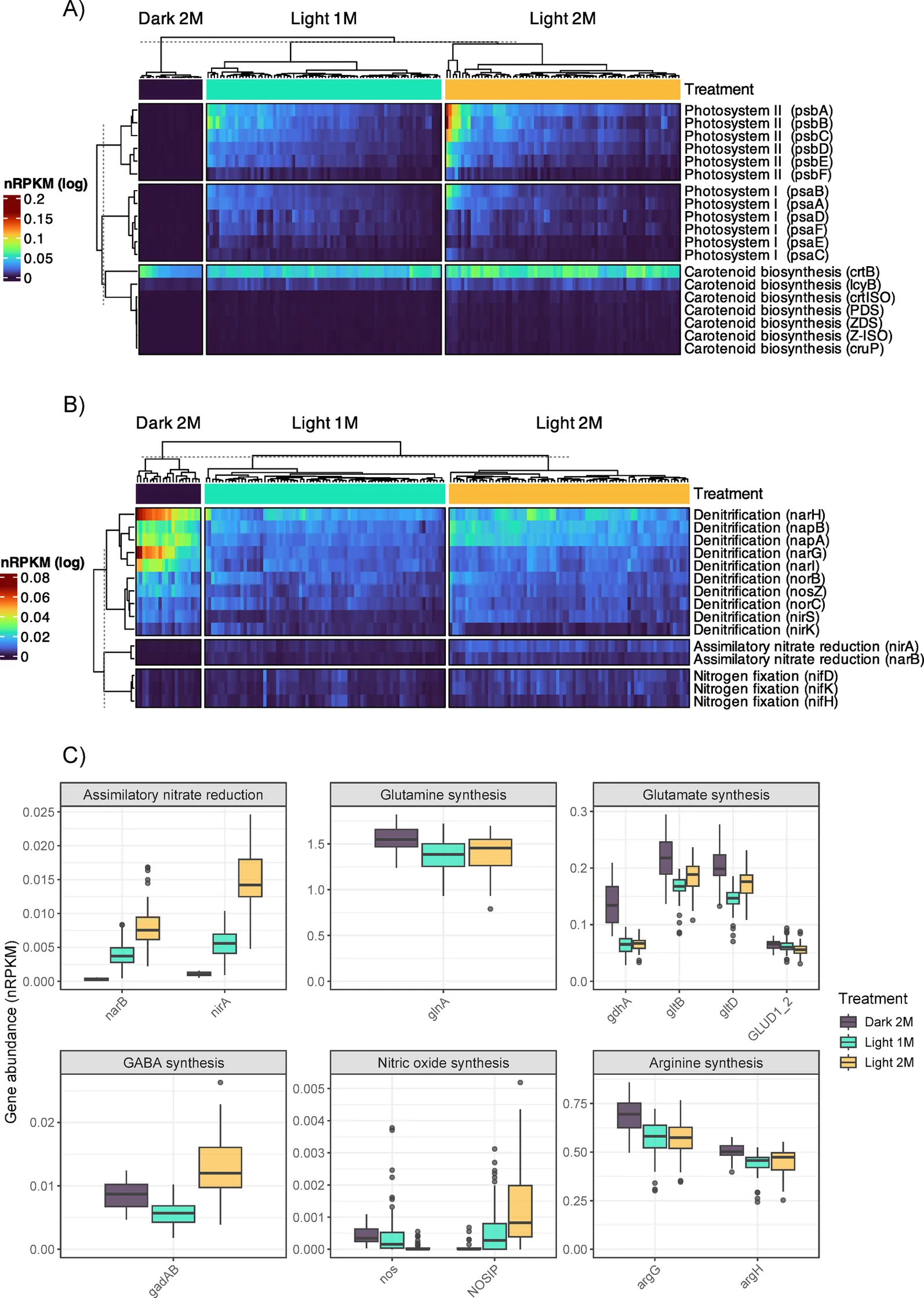
Abundance (nRPKM) of genes encoding photosystems and carotenoid biosynthesis (**A**), and denitrification, assimilatory nitrate reduction and nitrogen fixation (**B**). Heatmap columns represent biofilm samples and are grouped by treatment, while rows represent genes and are grouped by metabolic pathway. Rows and columns are hierarchically clustered by Euclidean distance and displayed as dendrograms. (**C**) Abundance (nRPKM) of genes encoding the biosynthesis of amino acids glutamine, glutamate and arginine, as well as genes encoding nitric oxide synthase (*nos*) and nitric oxide synthase interaction protein (*NOSIP).* nRPKM (log) indicates log10 + 1 transformation to improve visualisation. All genes were identified as differentially abundant among treatments based on multivariate linear models except *nos*, which did not meet abundance filtering thresholds

Given the pronounced differences in gene composition among treatments, we next sought to identify the major genes and pathways driving these patterns and determine which might have contributed to higher larval settlement observed on light biofilms compared to dark. Using multivariate linear models, we identified the 50 KOs most significantly enriched and the 50 most significantly reduced in light 2M biofilms compared to all other treatments (Figure S3). As expected, light biofilms were enriched in genes encoding the photosynthetic apparatus, which were absent from dark biofilms (Fig. 2A; Supplementary File 2). Analysis of MAGs confirmed these genes were primarily encoded in taxa classified as Cyanobacteria (Figure S4); however, it’s likely photosynthetic eukaryotes are also present within the biofilm. Conversely, dark biofilms were characterised by a 1.3-fold increase in the mean abundance of genes encoding the citrate (TCA) cycle compared to light 1M and 2M biofilms (Figures S3 & S5), suggesting increased aerobic metabolism. Although unlikely to directly affect larval settlement, shifts in carbon metabolism can influence biofilm structure and chemistry, with the potential for indirect consequences on settlement [63].

### Carotenoids and nitrate reduction may contribute to settlement induction on light biofilms

**Fig. 3:**
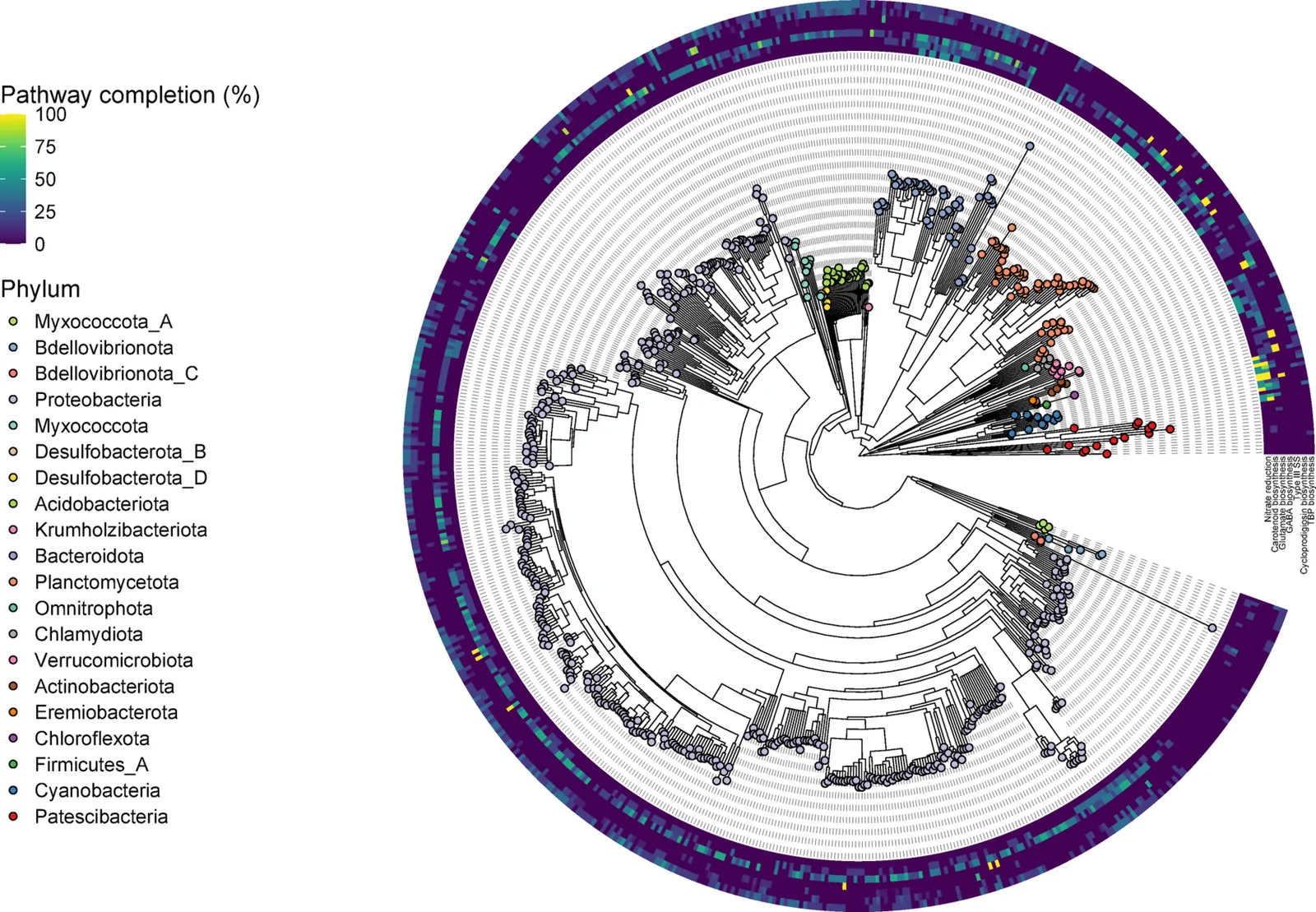
MAGs with potential settlement inducing or settlement enhancing functions. Branch tips of the phylogenomic tree are coloured by MAG phylum while outer ring heatmaps represent pathway completion for each metabolic pathway. Glutamate synthesis represents the proportion of the 4 biosynthesis genes that are encoded in each MAG. A high-resolution tree image has been provided in the supplementary information (Supplementary File 4) that includes tip labels representing MAG family level classification. Metabolic pathways from inner to outer ring: nitrate reduction, carotenoid biosynthesis (beta-carotene), glutamate biosynthesis, GABA biosynthesis, type III SS, cycloprodigiosin biosynthesis and TBP biosynthesis

Genes encoding carotenoid biosynthesis were nearly twice as abundant in light 2M biofilms compared to dark biofilms, particularly those for beta-carotene (Fig. 2A). While these genes can be involved in light capture within the photosynthetic apparatus, non-photosynthetic bacteria can possess them too. Carotenoids impart red/orange pigmentation, and coral larvae have been shown to be attracted to surfaces with these colours [64]. Furthermore, carotenoids can enhance larval metamorphosis in the presence of other inducers [65]. Genes in the beta-carotene biosynthesis pathway were widespread, with MAGs from seven phyla encoding lycopene cyclase (*lcyB*), which converts lycopene to beta-carotene (Fig. 3; Table S6; Supplementary File 3). These included families such as *Flavobacteriaceae* and *Sphingomonadaceae*, previously associated with high settlement biofilms [21, 23]. However, only Cyanobacteria MAGs encoded the complete or near complete (> 80%) beta-carotene biosynthesis pathway. Nonetheless, the presence of carotenoids in light conditioned biofilms may enhance settlement by attracting larvae to the biofilm surface and amplifying the effects of other inductive compounds.

Light biofilms were also enriched in genes encoding assimilatory nitrate reduction (*narb, nirA*; Fig. 2B), with light 2M biofilms showing a 17-fold increase in mean abundance compared to dark biofilms. This pathway reduces nitrate to ammonia, suggesting increased ammonia availability may support the synthesis of the amino acids glutamine and glutamate [66]. Genes encoding glutamine synthase (*glnA*), glutamate synthase (from glutamine; *gltB*, *gltD*) and glutamate dehydrogenase (*gdhA*, *GLUD1_2*) were present across all biofilm treatments, however their abundances were slightly higher with 1.1–twofold increase in dark biofilms compared to light 2M, except for glutamate dehydrogenase (*GLUD1_2*; Fig. 2C). Despite this, light 2M biofilms could still be enriched for the synthesis of glutamate through increased availability of ammonia. Since glutamate is the precursor of the neurotransmitter gamma-aminobutyric acid (GABA), this may have implications for increasing larval settlement (see section below). Here, we found that glutamate decarboxylase (*gadAB*), which catalyses the conversion of glutamate to GABA, showed a 1.5-fold increase in mean abundance in light 2M biofilms compared to dark, suggesting light 2M biofilms may be enriched for GABA production. Further analysis revealed diverse taxa across five phyla encoded either nitrate or nitrite reductase (*narB* and *nirA*, respectively), including families previously associated with high settlement such as *Flavobacteriaceae* and *Pirellulaceae* [23, 67]. However, only Cyanobacteria MAGs encoded the full assimilatory nitrate reduction pathway (Fig. 3; Table S6; Supplementary File 3). Genes for glutamine and glutamate synthesis were also widespread, with almost 600 MAGs encoding *glnA*, and over 400 encoding at least one of *gltB*, *gltD, gdhA* and *GLUD1_2* (Fig. 3; Figure S6). Taken together, these findings suggest that nitrogen assimilation in light treatment biofilms may enhance glutamine and glutamate synthesis, with potential to support increased synthesis of GABA.

### Increases in nitric oxide may inhibit settlement on dark biofilms

Analysis of nitrogen metabolism revealed that the mean abundance of genes involved in denitrification was more than twofold higher in dark biofilms compared to light 1M and 2M biofilms (Fig. 2B). In contrast, genes encoding nitrogen fixation were 1.8 and 2.8-fold higher in light 1M and 2M biofilms compared to dark (Fig. 2B). Together, these patterns suggest reduced nitrogen bioavailability in dark biofilms [68]. Denitrification involves reducing nitrite to nitric oxide (NO), and although NO is usually a short-lived intermediate, it may also be released as a by-product [69]. Further, the nitrite reductase genes *nirK* and *nirS* can occur in bacteria lacking complete denitrification pathways, suggesting nitrite can be reduced to NO independently of denitrification [68]. This is reflected in our results, where 28 MAGs encoded either *nirK* or *nirS*, while complete denitrification pathways were restricted to eight Proteobacteria MAGs, including *Rhodobacteraceae*, *Kiloniellaceae* and *Methyloligellaceae*, and one *Flavobacteriaceae* MAG (Figure S4; Table S6; Supplementary File 3). We also screened for nitric oxide synthase (*nos*) genes. Although rare and only found in a single Acidobacteriota MAG (*UBA5704*), *nos* had a nearly ninefold higher mean abundance in dark biofilms compared to light 2M (Fig. 2C), supporting elevated NO production in dark biofilms. Conversely, the gene encoding nitric oxide synthase-interacting protein (*NOSIP*) had a 13-fold increase in mean abundance in light 2M biofilms compared to dark (Fig. 2C). This protein regulates *nos* activity [70]*,* and its presence may indicate suppression of NO in light biofilms. However, *NOSIP* has not been characterised in prokaryotes and was not detected in any MAGs, suggesting it may originate from microbial eukaryotes such as fungi [71].

The production and regulation of NO have important implications for larval settlement. NO is a gaseous signalling molecule that is typically considered inhibitory; however, NO can be inductive for some species, such as the sponge *A. queenslandica* and the ascidian *H. momus* [72, 73]. While endogenous NO is primarily considered the cue for regulating settlement, larvae can respond to exogenous NO. For example, an exogenous NO donor induced settlement of sponge larvae in the absence of other cues [72], while exogenous NO donors inhibited settlement in mussel larvae [74]. Further, NO is a dispersal cue in a wide range of biofilms, including mixed-species communities from aquatic environments [75, 76]. Consequently, NO may have a negative impact on settlement through disrupting biofilm formation. Since dark biofilms have higher potential for producing larger amounts of unregulated NO, excess NO may be inhibiting larval settlement on these biofilms. If NO proved inhibitory in corals, settlement could potentially be enhanced in aquaculture by applying *nos* inhibitors to reduce NO production.

An important precursor of NO production via *nos* is the amino acid L-arginine, which can be acquired from the environment or bacterial symbionts [77, 78]. Arginine biosynthesis is common in bacteria, with over 400 MAGs encoding biosynthesis genes (*argGH*; Figure S6; Table S6; Supplementary File 3). However, we found that genes encoding L-arginine synthesis were 1.2-fold higher in mean abundance in dark biofilms compared to light 1M and 2M, indicating the potential for larger amounts of L-arginine in dark biofilms (Fig. 2C). This may further enhance NO production and subsequently inhibit settlement on these biofilms. For example, exposure to exogenous L-arginine upregulated *nos* expression in mussel larvae, leading to higher levels of NO and a decrease in settlement [79]. Taken together, NO likely has an important role in regulating coral larval settlement and biofilms may inhibit settlement through increased levels of exogenous NO or L-arginine.

### Gene associations with larval settlement inform putative mechanisms for induction and inhibition

To identify biofilm genes or pathways that may directly influence settlement independent of conditioning treatment, we examined associations between KO abundance and larval settlement for each coral species using multivariate linear models. Due to the large number of associated KOs, we reduced the number of significant KOs using sPLS to a subset of 193 that were significantly associated with settlement in at least one coral species (Figures S7-10). Many of these encoded intracellular metabolism unlikely to directly affect settlement, hence we focussed our efforts on genes that encoded bacterial surface structures, appendages or the production of molecules which could plausibly interact with larvae. The KOs identified across both analyses varied markedly among the different coral species, indicating that each species may respond uniquely to the complex metabolic interactions occurring within biofilms. This may reflect different ecological preferences among the diverse corals studied, or the variation in larval morphology and sensory capacity, which may underlie differential responses to settlement-inducing compounds [80]. However, when considering KO associations from linear models only, several KOs were consistently associated with settlement across multiple coral species. We therefore used the sPLS-reduced set to identify candidate genes of interest, then checked whether those genes were also associated with settlement in other coral species based on linear models.

### GABA may be an inductive cue for coral larvae

**Fig. 4:**
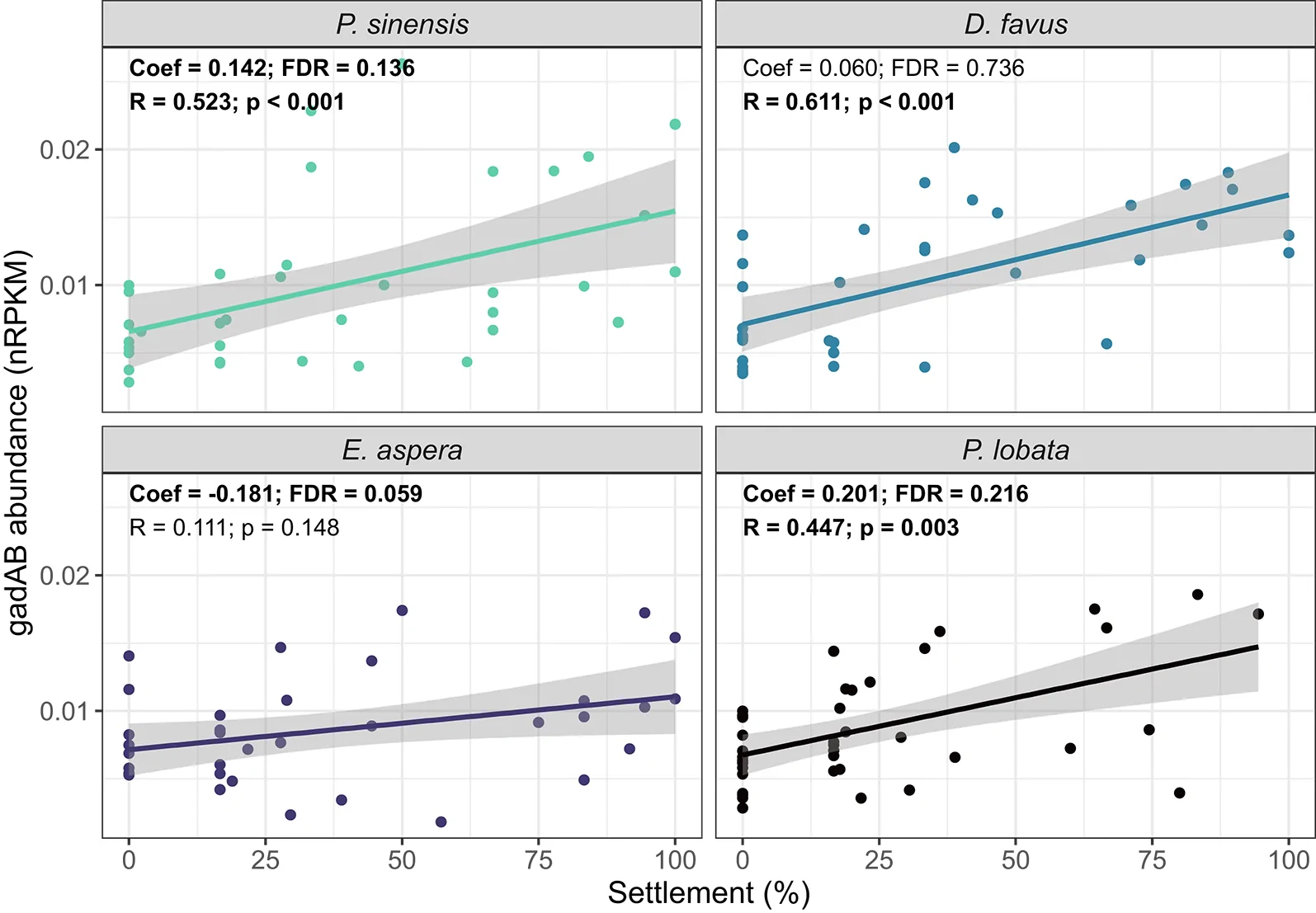
The relationship between *gadAB* abundance (nRPKM) and coral larval settlement (%). Each point represents a biofilm sample and plots are grouped by coral species. A linear regression line is shown as the line of best fit. Coef and FDR refer to the model coefficient (effect size) and false discovery rate from multivariate linear models. R and p refer to the Pearson correlation coefficient and associated p-value. Values in bold indicate significant results

The gene encoding glutamate decarboxylase (*gadAB*) showed a positive association with larval settlement of *P. lobata* and *P. sinensis*, while *D. favus* showed no significant relationship and *E. aspera* had a negative association (Fig. 4; Table S7)*.* However, when considering the Pearson correlation coefficient (which does not incorporate covariates), the abundance of *gadAB* was positively correlated with *D. favus* settlement while there was no significant relationship with *E. aspera* settlement (Fig. 4). Hence, not only were pathways for GABA production enriched in 2M light biofilms, the key enzyme for GABA production also shows a direct association with settlement. Further, *gadAB* was encoded in 20 MAGs across five phyla (Fig. 3; Table S6; Supplementary File 3), including families previously linked to settlement induction such as *Pirellulaceae*, *Rhizobiaceae* and *Rhodobacteraceae* [23, 67]. GABA has been shown to induce settlement of a variety of marine invertebrate larvae such as mussels, oysters and abalone [81, 82]. Although its direct role in coral larval settlement has not been tested, its precursor glutamic acid induced low levels of settlement of *Leptastrea purpurea* larvae [83]. Furthermore, GABA receptors in *Acropora millepora* larvae were upregulated during settlement [84], while upregulation of GABA receptors stopped during metamorphosis stage of *Acropora tenuis* settlement, suggesting GABA receptors are active during the searching and attachment phase [85]. Overall, these results suggest that GABA may act as an inductive neurotransmitter in coral larval settlement, potentially synthesised and supplied by bacteria in marine biofilms [86, 87]. Experimental validation of GABA’s role in coral larval settlement would be valuable for future research.

### Secretion systems may have positive and negative effects on settlement

**Fig. 5:**
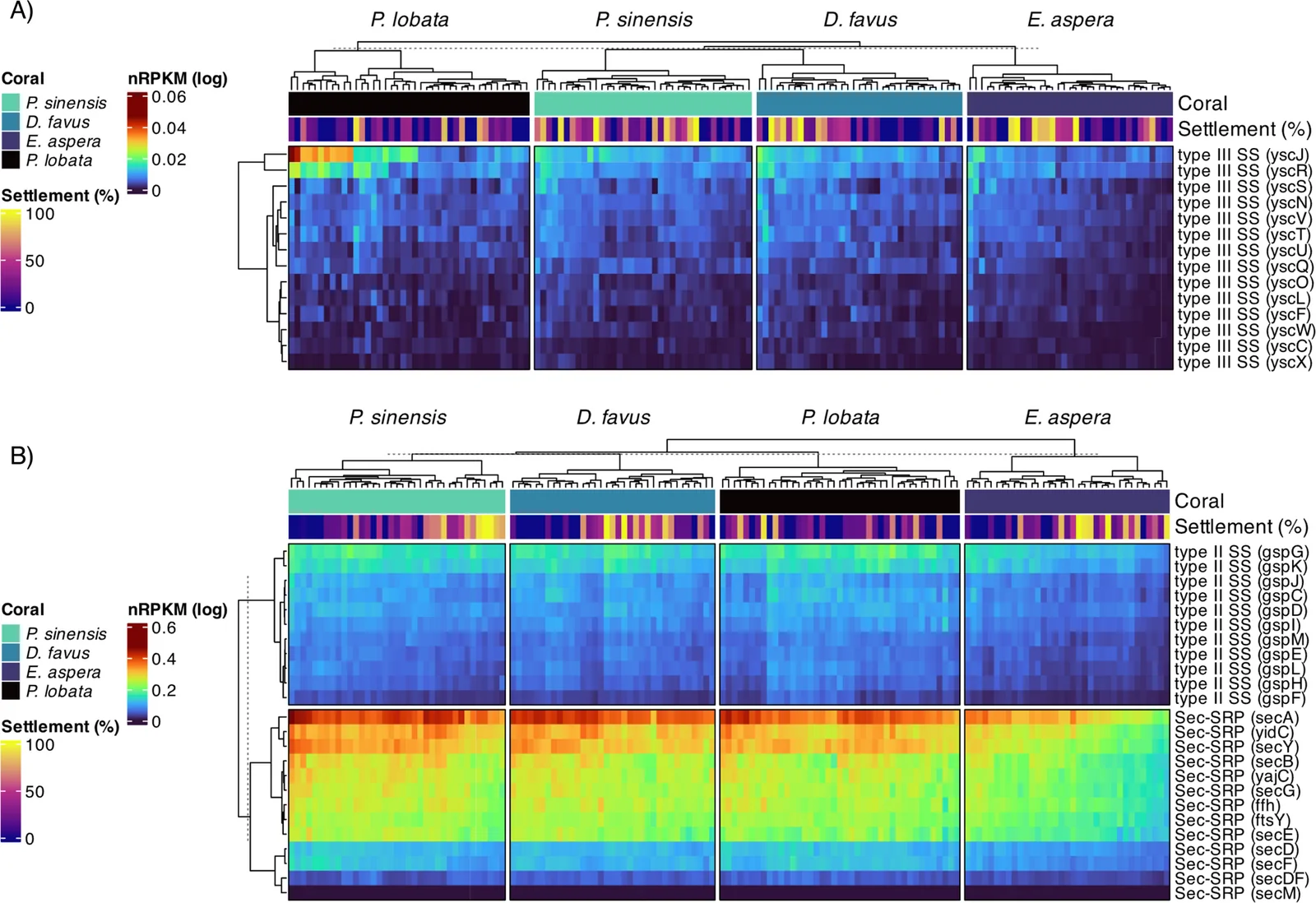
Abundance (nRPKM) of genes encoding the type III secretion system (**A**), type II secretion system and the sec-SRP pathway (**B**). Columns represent each biofilm sample and are grouped by coral species, rows represent each gene and are grouped by secretion pathway. Rows and columns are hierarchically clustered by Euclidean distance and displayed as dendrograms. Top colour bar indicates coral species, while the second colour bar represents the proportion of larval settlement in each biofilm sample. nRPKM values were log + 1 transformed to improve visualisation. For the type III SS, genes *yscF*, *yscU* and *yscX* had a positive association with settlement in at least one coral based on multivariate linear models. For the type II SS and Sec-SRP pathway, all genes shown had a negative association with settlement in at least one coral

A subset of genes encoding the type III secretion system (SS) were positively associated with *P. sinensis* and *P. lobata* settlement (*yscF, yscU* & *yscX*), whereas all other significant associations with secretion system genes were negative (Fig. 5A; Table S7). Similarly, genes encoding the needle of the type III SS (*yscF*, *yscO*, *yscX*) were more abundant in light 2M biofilms compared to dark, while most other genes encoding secretion systems were more abundant in dark biofilms compared to light (Table S7). Type III SS are used by some bacteria to deliver effector proteins into host cells [88], and a similar contractile injection system in *Pseudoalteromonas luteoviolaceae* induces metamorphosis of the tube worm *Hydroides elegans* [6]. A comparable mechanism may exist for corals, or the type III SS could instead facilitate biofilm traits that are attractive to coral larvae. For example, the type III SS can promote cell aggregation through the release of a host factor [89], or suppress growth of protozoans [90]. While not all genes were found, MAGs from four phyla encoded 53–87% of type III SS genes, including families *Cellvibrionaceae*, *Pirellulaceae* and *Arenicellaceae* (Figure S6; Table S6; Supplementary File 3).

In contrast, a subset of genes encoding type I, II, IV and VI SS, as well as the sec-SRP pathway, were negatively associated with larval settlement (Fig. 5B; Table S7). In particular, most type II SS genes were present and negatively associated with settlement of *P. sinensis* and *E. aspera*, while genes encoding the sec-SRP pathway were negatively associated with *P. sinensis, E. aspera* and *D. favus* settlement*.* The sec-SRP pathway is found in most bacteria and transports proteins across the cytoplasmic membrane, some of which may be secreted outside the cell via the type II SS [91]. Similarly, the type II SS is common in Gram-negative bacteria and translocates a wide range of proteins, including enzymes and toxins, from the periplasm to the outer membrane or extracellular environment [92]. Both the type II SS and sec-SRP genes were widespread among taxa in this study, with 128 MAGs across 7 phyla encoding at least 50% of the type II SS genes, and 466 MAGs across 16 phyla encoding at least 50% of the sec-SRP pathway (Figure S7; Table S6; Supplementary File 3). Although common, multiple gene copies of these pathways may reflect high secretory loads [93, 94]. Furthermore, the type II SS may directly inhibit settlement in some taxa. For example, *Pseudoalteromonas* sp. sf57 inhibits settlement of *H. elegans*, yet mutant strains lacking a type II SS gene (*gspD*) became inductive, showing increased biofilm density and the loss of the inhibitory compound [95]. Hence, bacterial secretion systems may both promote and inhibit larval settlement depending on the molecules released.

### Flagella and LPS have negative associations with settlement

**Fig. 6:**
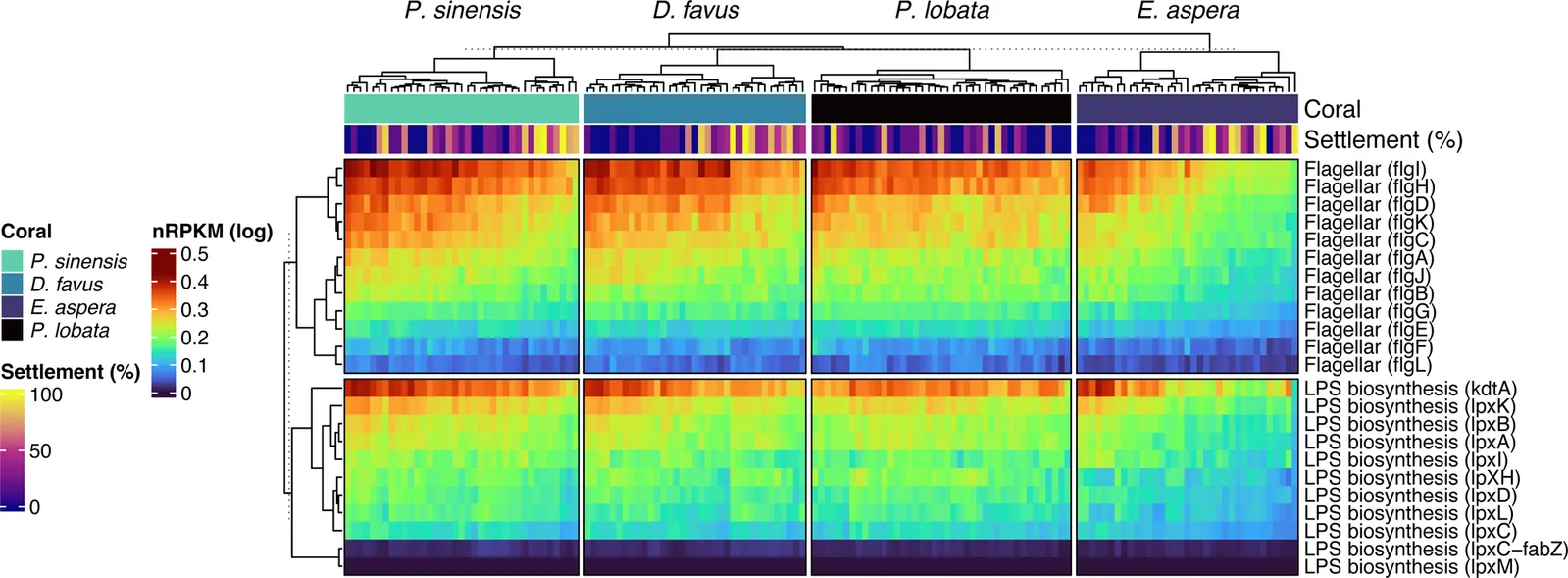
Abundance (nRPKM) of genes encoding the flagellar basal body and hook protein and genes encoding the lipopolysaccharide biosynthesis pathway for the lipid-A component. Columns represent each biofilm sample and are grouped by coral species, rows represent each gene and are grouped by structure type. Rows and columns are hierarchically clustered by Euclidean distance and displayed as dendrograms. Top colour bar indicates coral species, while the second colour bar represents the proportion of larval settlement in each biofilm sample. nRPKM values were log + 1 transformed to improve visualisation. All genes shown were negatively associated with settlement in at least one coral based on multivariate linear models, except *flgF.* Genes *flgD, flgE, flgK* and *flgL* encode the hook and hook-associated components, while the remaining genes are involved in the basal body assembly

Bacterial flagella are used for motility with roles in early biofilm development and pathogenicity [96]. Our results show a negative association between settlement of *P. sinensis* and *E. aspera* and the abundance of genes encoding the flagellar hook protein and basal body (Fig. 6; Table S7). During biofilm formation, motile bacteria are known to transition toward a sessile state in which motility becomes less important [96]. Similarly, non-motile, slow-growing bacteria have been observed to increase in relative abundance as biofilms mature [97]. Hence, these negative associations may indicate early-stage biofilms with more motile bacteria that are less attractive to coral larvae [61]. Consistent with this, we found that 1M light biofilms encoded a greater number of flagellar genes than 2M light biofilms (Table S7). Flagella may also directly influence settlement. For example, flagellar protein extracts from *Pseudoalteromonas marina* induced settlement of the mussel *Mytilus coruscus* [98]. Although contrasting with our negative associations, this illustrates a potential direct role of flagella in larval settlement.

Lipopolysaccharides (LPS) are a large and diverse family of molecules found in the outer membrane of Gram-negative bacteria [99], and genes encoding LPS biosynthesis were negatively associated with settlement for all corals except *P. lobata* (Fig. 6; Table S7). LPS is a primary bacterial molecule that animals can perceive through a range of receptors that can illicit both positive and negative responses [100]. For example, LPS may trigger an immune response to potential infections in coral and other animals [101, 102], but also plays a role in establishing mutualistic symbioses between bacteria and hosts [103, 104]. Its effect on larval settlement is therefore likely variable. For example, LPS from inductive bacteria can stimulate settlement in the tubeworm *H. elegans*, whereas LPS from non-inductive strains had no effect [105]. The negative associations observed here may reflect a predominance of inhibitory or neutral LPS-producing bacteria within biofilms. Alternatively, a reduction in LPS biosynthesis genes in high settlement samples may represent a community shift towards taxa with reduced LPS biosynthesis pathways. This can be seen in 2M light biofilms, which encoded fewer LPS genes compared to 1M light biofilms (Table S7). Given this versatility, future studies should examine how structural or compositional differences in LPS from inductive and inhibitory bacteria influence coral larval settlement.

### Additional genes of interest: cycloprodigiosin and tetrabromopyrrole (TBP) biosynthesis

Cycloprodigiosin has recently been identified as a settlement inducer for both brooding and broadcast-spawning coral species [106]. It was therefore unexpected that genes encoding alkylglycerol monooxygenase (*AGMO*) showed negative associations with settlement in *P. sinensis* and *E. aspera* (Figure S11; Table S7). *AGMO* is part of the fatty acid hydroxylase protein family and is the closest characterised homologue of the gene *PRUB680*, which causes cyclisation of prodigiosin to cycloprodigiosin [53]. While *AGMO* is not known to be functional in bacteria, we identified 239 MAGs encoding this gene (Figure S6; Table S6; Supplementary File 3), suggesting that genes annotated as *AGMO* could represent the bacterial homologue *PRUB680* [53]. To this end, we looked at the phylogeny of genes annotated as *AGMO* in our biofilms along with the *PRUB680* gene and a curated set of fatty acid hydroxylases. *AGMO* genes from this study clustered in two major clades, with the larger clade including the gene *PRUB680* (Figure S12). However, this clade shows high sequence variability, and given the broad functional diversity of fatty acid hydroxylases, not all genes annotated as *AGMO* are likely to represent functional homologues of *PRUB680*.

The presence of cycloprodigiosin biosynthesis genes was investigated further using a translated nucleotide BLAST search, along with TBP biosynthesis genes due to its characterised role in inducing coral larval settlement [15]. Based on conservative matches of homology (e-value < 1e−10; bit score > 100), we identified 174 MAGs encoding the gene *PRUB680*, of which 149 MAGs encoded additional genes for prodigiosin biosynthesis, though pathways were incomplete (18–45%; Fig. 3). By contrast, when considering annotations based on the KEGG database, only one uncharacterised Myxococcota MAG encoded an additional two genes (*pigA and pigI*) for prodigiosin biosynthesis, and hence this pathway was incomplete (Table S6; Supplementary File 3). Together, these results suggest it is unlikely that MAGs encoding *AGMO*/*PRUB680* homologues are producers of cycloprodigiosin. Similarly, we found 417 MAGs encoding at least one gene required for TBP biosynthesis, however only 13 MAGs encoded pathways that were ≥ 50% complete (Fig. 3; Table S6; Supplementary File 3), including taxa such as *Cellvibrionaceae*, *Rhodobacteraceae* and *Sphingomonadaceae*. Incomplete biosynthesis pathways suggest these compounds are unlikely to be responsible for settlement induction in these biofilms, highlighting the potential for additional settlement cues that are yet to be discovered.

### A proposed model of biofilm-induced coral larval settlement

**Fig. 7:**
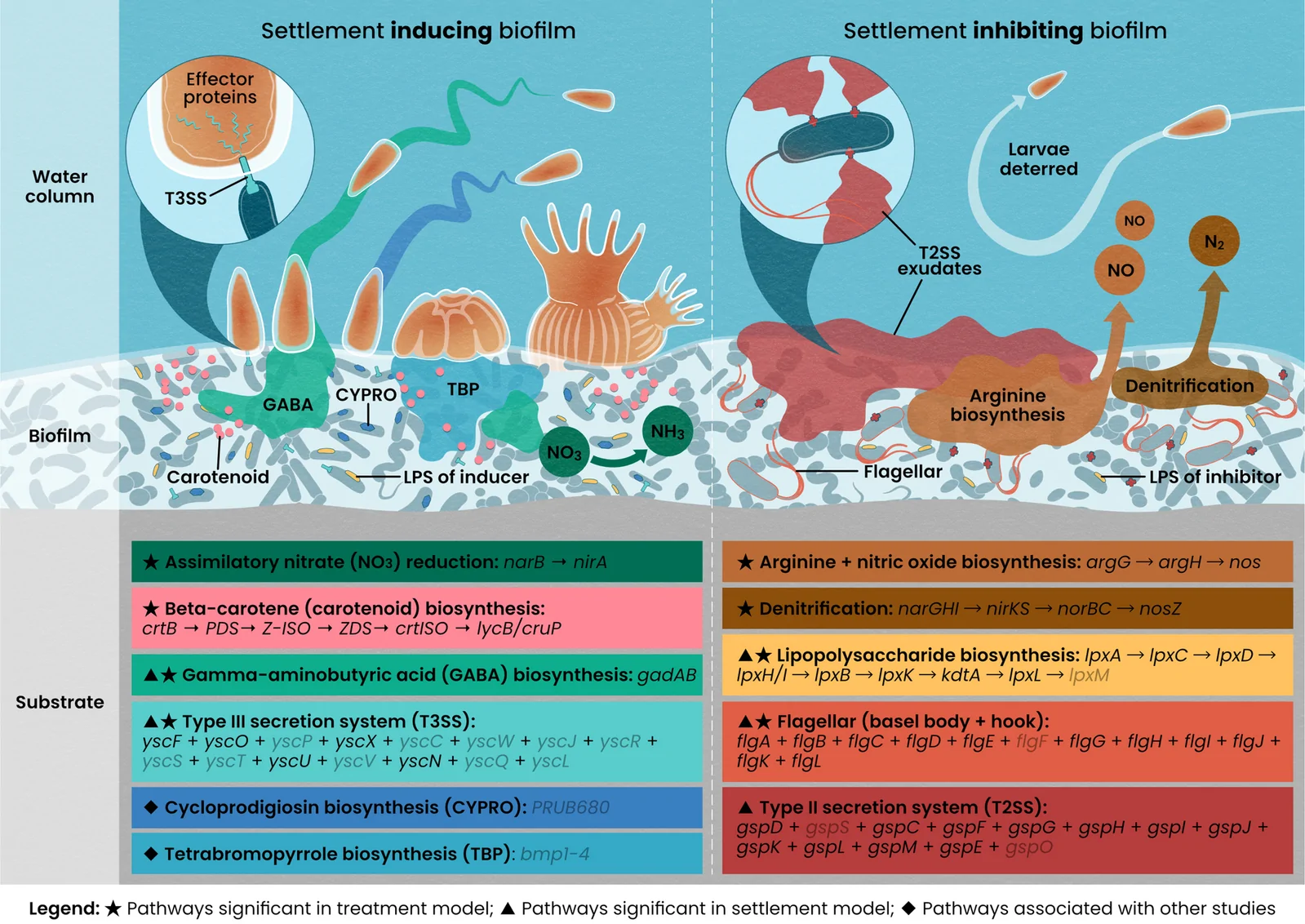
Overview of potential settlement inducing and inhibiting properties by bacteria within marine biofilms. Genes that encode each metabolic pathway or bacterial structure are shown beneath and genes in bold were found to be significantly associated with settlement. Symbols next to pathways denote in which analysis genes were found

Our analysis of biofilms associated with high and low coral larval settlement suggests broad ecological trends (Fig. 7). Larvae may be attracted to biofilms enriched in pigmented compounds such as carotenoids, while biofilms with high nitrogen assimilation may produce more inductive cues. Conversely, biofilms with elevated NO or its precursor arginine likely inhibit larval settlement. Once larvae contact the biofilm, neuropeptides such as GABA may encourage searching and attachment, while strong cues for metamorphosis may include secondary metabolites such as TBP and cycloprodigiosin or effector proteins delivered through a type III SS. Conversely, larvae may actively avoid unfavourable areas, signalled by toxins secreted via the type II SS, an abundance of flagella indicating less established biofilms, or LPS signalling potential infection (Fig. 7). Importantly, several mechanisms may be either inductive or inhibitory depending on the taxa involved. For example, LPS from inductive bacteria may promote settlement, whereas LPS from pathogens likely suppress it. As metagenomic data describes only metabolic potential, future research would benefit from testing these hypotheses experimentally. Promising approaches include metatranscriptomics to assess gene expression in the biofilm during settlement, metabolomics to identify which compounds are produced, and genetic manipulation of bacterial isolates encoding candidate mechanisms of induction [107].

Biofilms are ubiquitous on marine surfaces and play critical roles in the settlement of marine invertebrate larvae [5]. Our study applied a metagenomic approach to identify putative bacterial mechanisms of induction that could be harnessed to improve settlement in aquaculture. For example, settlement could potentially be enhanced with bacterial-derived chemical cues such as GABA or manipulating biofilms to favour characteristics such as low NO production or high nitrogen assimilation. Additionally, coral larvae showed species-specific settlement associations with certain biofilm genes. This may correspond to different settlement preferences in the wild and could be leveraged to developed inductive cues tailored to enhance settlement of difficult-to-settle non-acroporid species. Together, our findings provide a comprehensive overview of how biofilms can both induce and inhibit coral larval settlement. They underscore the complexity of larval-biofilm interactions, where multiple mechanisms operate simultaneously to guide larvae toward optimal settlement sites.

## Supplementary Information

Additional file 1.

Additional file 2.

Additional file 3.

Additional file 4.

Additional file 5.

Additional file 6.

## Data Availability

All sequence data analysed during this study are available at NCBI (https://www.ncbi.nlm.nih.gov) under the BioProject ID PRJNA1314392 with the accession numbers SRR35232105–SRR35232292, while MAGs are available at http://data.qld.edu.au/public/Q8887. Scripts for calculating protein abundance are available at https://github.com/julianzaugg/protein_abundance_rpkm_singlem and functions for parsing singleM output files are found at https://github.com/julianzaugg/singlem_output_process. All other code used for the analyses are available at https://github.com/paobrien/Mechanisms-of-coral-settlement.
