# Supplementary material for: Metagenomic insights into mechanisms of coral larval settlement induction and inhibition by marine biofilms
Source: Environ Microbiome. 2026 Aug 23;21:105. doi: 10.1186/s40793-026-00945-0 (PMC13543475; doi:10.1186/s40793-026-00945-0)
Supplement: Supplementary file 1 [file 40793_2026_945_MOESM1_ESM.docx]

**Metagenomic insights into mechanisms of coral larval settlement induction and inhibition by marine biofilms**

Paul A. O’Brien^1*^, Sara C. Bell^2^, Andrew P. Negri^2^, Shannon R. Kjeldsen^2,3^, Julian Zaugg^1^, Nicole S. Webster^1,4^, Muhammad Abdul Wahab^2^, Inka Vanwonterghem^1,5†^, Laura Rix^1†^

**Supplemental Figures**

**Figure S1.** Relative abundance of the top 20 most abundant prokaryotic phyla based on SCM genes. Dominant taxa were calculated by the total sum of relative abundance across all samples. Non-dominant taxa were grouped as ‘Other’. All phyla except Thermoproteota and Firmicutes were represented in MAGs.

**Figure S2.** Relative abundance of the top 20 most abundant prokaryotic families based on SCM genes. Dominant taxa were calculated by the total sum of relative abundance across all samples. Non-dominant taxa were grouped as ‘Other’. All families were represented in MAGs

A)

B)

**Figure S3.** Top 50 KOs more abundant in light 2M (**A**), or less abundant in light 2M (**B**), compared to other treatments based on multivariate linear models. Heatmaps are coloured by KO abundance (nRPKM). Columns represent each biofilm sample and are grouped by treatment, while rows represent each KO and are grouped by pathway as described in the R package ‘KEGGREST’, denoted by row labels. Rows and columns are hierarchically clustered by Euclidean distance and displayed as dendrograms. For instances where KOs are present in multiple pathways, labels have been shortened to pathways of interest for readability.

**Figure S4.**  MAGs with potential for carbon and nitrogen metabolism. Branch tips of the phylogenomic tree are coloured by MAG phylum while tip labels represent MAG family level classification. Outer ring heatmaps represent pathway completion for each metabolic pathway, where the TCA cycle and Calvin cycle represent the proportion of steps found, while the remainder represent the proportion of genes found.

**Figure S5.** Abundance (nRPKM) of genes involved in the TCA cycle and Calvin cycle across different biofilm treatments. Columns represent biofilm samples while rows represent genes. Biofilm samples are grouped by conditioning treatment, while genes are grouped by metabolic pathway. Rows and columns are hierarchically clustered by Euclidean distance and displayed as dendrograms. nRPKM values were log10+1 transformed to improve visualisation. All genes were more abundant in dark biofilms compared to light 2M based on multivariate linear models, except LSC2, aarC/cat1, MDH1, MDH2 (TCA cycle), and rbcL, rbcS, fbp3, E3.1.3.37 (Calvin cycle).

**Figure S6.** MAGs encoding biosynthesis genes for amino acids (glutamine, glutamate and arginine), gamma-aminobutyric acid (GABA), alkylglycerol monooxygenase (AGMO), the sec-SRP pathway, type II and III secretion systems (SS), flagella and lipopolysaccharide biosynthesis. Branch tips of the phylogenomic tree are coloured by MAG phylum while tip labels represent MAG family level classification. Outer ring heatmaps represent pathway completion for each biosynthesis pathway.

S7) *Platygyra sinensis*

S8) *Dipsastrea favus*

S9) *Echinophyllia aspera*

S10) *Porites lobata*

**Figures S7-10.** Genes associated with settlement based on multivariate linear models and sPLS analysis for *P. sinensis* (**S7**), *D. favus* (**S8**), *E. aspera* (**S9**) and *P. lobata* (**S10**). Columns represent biofilm samples and are grouped by settlement categories; None (0% settlement), Low (≤ 50%) and High (> 50%). Rows represent each KO (shown as *KO: gene name*) and are partitioned into two groups based on abundance (k-means = 2). Top colour bar represents conditioning duration (1 month or 2 months), and second colour bar shows proportion of corals settled. Within groups, rows and columns are hierarchically clustered by Euclidean distance and displayed as dendrograms. nRPKM (log) indicates log10+1 transformation to improve visualisation.

**Figure S11.** The relationship between *AGMO* abundance (nRPKM) and coral larval settlement (%). Each point represents a biofilm sample and plots are grouped by coral species. A linear regression line is shown as the line of best fit. Coef and FDR refer to the model coefficient (effect size) and false discovery rate from multivariate linear models. R and p refer to the Pearson correlation coefficient and associated p-value. Values in bold indicate significant results

**Figure S12**. Gene tree for AGMOs found in this study (purples tip points), a curated set of fatty acid hydroxylases within the InterPro database (green tip points), and the PRUB680 gene responsible for cyclisation of prodigiosin (yellow tip point)
