# Supplementary material for: Metagenomic insights into mechanisms of coral larval settlement induction and inhibition by marine biofilms
Source: Environ Microbiome. 2026 Aug 23;21:105. doi: 10.1186/s40793-026-00945-0 (PMC13543475; doi:10.1186/s40793-026-00945-0)
Supplement: Supplementary file 2 [file 40793_2026_945_MOESM2_ESM.docx]

**Metagenomic insights into mechanisms of coral larval settlement induction and inhibition by marine biofilms**

Paul A. O’Brien^1*^, Sara C. Bell^2^, Andrew P. Negri^2^, Shannon R. Kjeldsen^2,3^, Julian Zaugg^1^, Nicole S. Webster^1,4^, Muhammad Abdul Wahab^2^, Inka Vanwonterghem^1,5†^, Laura Rix^1†^

**Supplementary file 1 – Methods**

**DNA extraction**

Biofilms on settlement tabs were thawed on ice and individual tabs placed into 50 mL centrifuge tubes with 5 mL of cell separation buffer containing 0.35% PVP, 0.5M sodium pyrophosphate and 0.5% Tween 20 in artificial sea water. Settlement tabs were then incubated at room temperature for 30 min with gentle rotation, followed by sonication for 10 min at 40KHz to dissociate the biofilm using a sonication bath. The supernatant was centrifuged at 300 x g for 5 minutes to pellet any debris from the settlement tab along with eukaryote cells and the supernatant recovered, with a 1 mL aliquot taken for 16S rRNA analysis (O’Brien et al., 2025). Supernatants for each metagenome extraction were then pooled (Table S1) by passing through a 100 µm cell strainer into a fresh 50 mL centrifuge tube. Each sample was then sequentially filtered through an 8 µm and 5 µm filter to remove any remaining eukaryote cells. Finally, the supernatant was filtered through a 0.22 µm sterivex filter to capture the enriched prokaryote cells, snap frozen in liquid nitrogen and stored at -80ºC until DNA extraction.

Sterivex filters were thawed on ice and 1.8 mL of filter-sterilised lysis buffer containing 50 mM Tris-HCL (pH 8.0), 40 mM EDTA (pH 8.0), 256 mg/ml sucrose and 18 µl of Lysozyme (100 mg/mL) added to the filter cartridge. Filters were incubated at 37° C for 1 hr with gentle agitation, followed by the addition 20 µL of Proteinase K/10% SDS solution (20 mg / mL) and incubation at 55° C for 1 hr with gentle agitation. The resulting lysate was recovered from the filter into a 5 mL microfuge tube and an equal volume of phenol:chloroform:isoamyl alcohol (25:24:1) added and mixed by inversion. Samples were centrifuged at 16,000 x g for 10 min and the top aqueous phase recovered into a new microfuge tube. Equal volumes of Chloroform:Isoamyl alcohol (24:1) were added, mixed by inversion and samples centrifuged for 16,000 x g for 10 min. The top aqueous phase was recovered into a new microfuge tube and DNA was precipitated by adding 0.8 volume of Isopropanol and incubation at room temperature for 15 min. The sample was then centrifuged at 20,000 x g for 25 min at 4°C and the supernatant removed. DNA was purified by adding 500 µL of 70% ethanol to the DNA pellet and centrifuging for 10 min at 20,000 x g and removing the supernatant. A second ethanol wash was performed as previously, and any remaining ethanol was evaporated for 20 min at room temperature. Finally, DNA was eluted in 25 µl of UltraPure DNase/RNase free distilled water at 4°C overnight and quantified using a Qubit fluorometer.
