# Supplementary material for: Metagenomic insights into mechanisms of coral larval settlement induction and inhibition by marine biofilms
Source: Environ Microbiome. 2026 Aug 23;21:105. doi: 10.1186/s40793-026-00945-0 (PMC13543475; doi:10.1186/s40793-026-00945-0)
Supplement: Supplementary file 3 [file 40793_2026_945_MOESM3_ESM.docx]

**Metagenomic insights into mechanisms of coral larval settlement induction and inhibition by marine biofilms**

Paul A. O’Brien^1*^, Sara C. Bell^2^, Andrew P. Negri^2^, Shannon R. Kjeldsen^2,3^, Julian Zaugg^1^, Nicole S. Webster^1,4^, Muhammad Abdul Wahab^2^, Inka Vanwonterghem^1,5†^, Laura Rix^1†^

**Supplementary file 2 – Results**

*Taxonomic comparisons among biofilm treatments*

SCM genes showed a higher abundance of Bacteroidota and Cyanobacteria in light 1M (6.6 ± 2.8% & 1.4 ± 0.8%) and light 2M (4.7 ± 1.9% & 1.9 ± 0.8%) treatment biofilms compared to dark treatments (1.1 ± 0.4% & 0.1 ± 0.07%). In contrast, Acidobacteriota was more abundant in dark biofilms (3.4 ± 0.9%) compared to light 1M (1.6 ± 1.1%) and light 2M (1.3 ± 0.8%) biofilms (Figure S1). At family level classifications, *Flavobacteriaceae* were more abundant in light 1M (1.3 ± 0.6%) and light 2M (1.5 ± 0.7%) biofilms compared to dark (0.7 ± 0.4%), while *Arenicellaceae* was more abundant in dark (1.9 ± 1.1%) biofilms compared to light 1M (0.1 ± 0.1%) and light 2M (0.1 ± 0.1%) biofilms (Figure S2). Additionally, these results show that a higher diversity of taxa was captured when using SCM genes compared to MAGs, likely due to the classification of low abundance sequences that aren’t assembled and binned. However, the number of taxa is potentially inflated due to the difficulty in classifying short reads. Nonetheless, these results show the dominant phyla and families are present across all biofilm treatments but differ in their abundances

*Carbon metabolism*

Since the Calvin cycle is typically associated with carbon fixation during photosynthesis, we searched for genes encoding this pathway within our biofilms. We found genes encoding the Calvin cycle in both light and dark treatment biofilms (Figure S6), suggesting carbon fixation is occurring through both photosynthesis and chemosynthesis (Hudson, 2024), with pathways present in Cyanobacteria, Alphaproteobacteria and Gammaproteobacteria MAGs (Figure S5). We also searched for MAGs encoding the citrate (TCA) cycle, given the higher abundance of genes in dark treatment biofilms. Partially complete pathways (>70%) were recovered from over 400 MAGs (Figure S5), including key genes encoding the enzymes citrate synthase and succinate dehydrogenase (Fuchs, 2011).
